# Epilepsy in Chinese Children With Mowat–Wilson Syndrome: Two Case Reports and Literature Review

**DOI:** 10.1111/jpc.70373

**Published:** 2026-03-27

**Authors:** Xuelin Huang, Dongling Yang, Jinqiu Wang, Qingqing Zhao, Yuyi Chen, Fengping Wei, Yiyan Ruan

**Affiliations:** ^1^ Department of Pediatric Neurology Maternal and Child Health Hospital of Guangxi Zhuang Autonomous Region, Guangxi Clinical Research Center for Pediatric Diseases Nanning China

**Keywords:** children, epilepsy, gene mutation, Mowat–Wilson syndrome, *ZEB2* gene

## 1 Introduction

Mowat–Wilson syndrome (MWS) is a rare autosomal dominant condition causing intellectual disability and congenital anomalies across multiple systems. It is caused by mutations in the zinc finger E‐box‐binding homeobox 2 gene (*ZEB2*). The *ZEB2* gene plays a significant role in embryonic development, particularly in the formation of the neural tube and neural crest [1, 2]. Studies indicate that approximately 75%–80% of MWS patients develop epilepsy [1, 3]. In this study, we retrospectively analysed the clinical features of two MWS children with epilepsy and assessed the findings from previously reported cases in order to enhance our clinical understanding of this condition.

## 2 Case Presentation

### 2.1 Case 1

**FIGURE 1:**
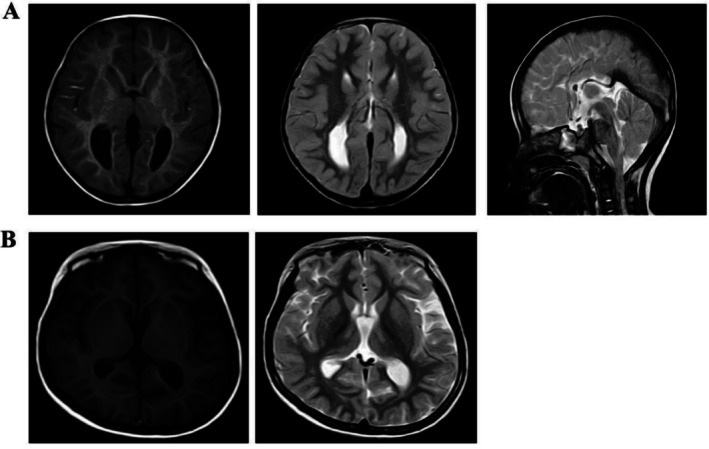
(A) An MRI of the brain of Case 1 showing congenital absence of a corpus callosum. (B) A brain MRI of Case 2 suggests cerebral atrophy and dilated supratentorial ventricles.

**FIGURE 2:**
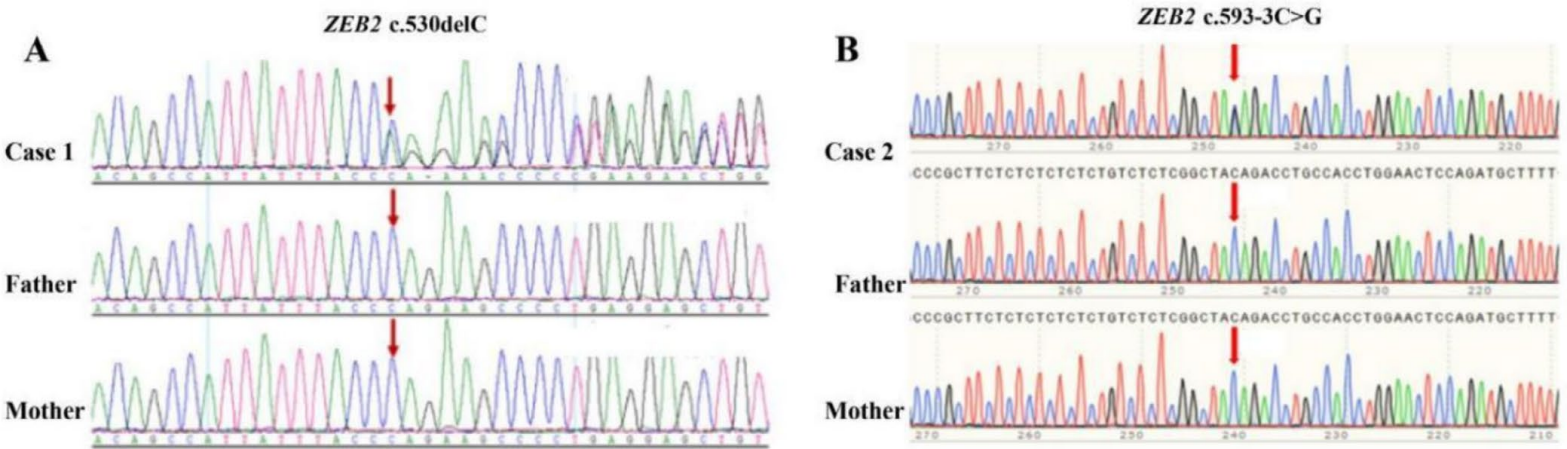
Sanger sequencing results of the two cases with *ZEB2* variants (A and B refer to Case 1 and 2, respectively).

A girl initially presented at our hospital when she was 5 months old due to unstable head control and inability to roll over. Gesell's developmental schedules showed a moderate developmental delay. Physical examination demonstrated wide‐set eyes, micrognathia, microcephaly, bilateral single transverse palmar creases, generalised hypotonia and poor responsiveness. At 9 months old, she had unprovoked seizures, followed by eye‐staring, limb rigidity and responsiveness. Her seizures occurred in clusters of one to three a day. Metabolic screening and routine biochemical examinations were unremarkable. Brain MRI revealed a congenital absence of a corpus callosum (Figure 1A). EEG demonstrated frequent generalised and multifocal spike waves as well as polyspike and slow‐wave discharges. Adrenocorticotropic hormone (ACTH) and topiramate (TPM) administration led to seizure remission for 1 year, and her parents discontinued TPM by themselves at the age of 3. However, 2 months after TPM withdrawal, seizures were induced after fever, and these manifested as left limb tonic–clonic, facial twitching and upward staring of her eyes for 20–30 min at a frequency of one to two times per month, whereby TPM was re‐initiated. At 4.5 years old, she presented with short stature, microcephaly and constipation. Her EEG detected an electrical seizure from the right occipital region during sleep. At her last follow‐up, conducted after a 4‐month seizure‐free period, the seizures recurred. A repeat EEG demonstrated focal and diffuse slow waves and spike‐and‐wave complexes, as well as polyspike‐and‐wave discharges, predominantly in the anterior and posterior head regions, with marked activation during sleep (Figure S1). A heterozygous c.530delC (p.Pro177fs*34) variant in Exon 5 of *ZEB2* (NM_014793.3) was detected in Case 1. Sanger sequencing of the proband's parents confirmed that neither of them carried this variant, indicating a de novo origin (Figure 2A). According to the American College of Medical Genetics and Genomics (ACMG) guidelines, this variant was classified as pathogenic (PVS1 + PS2 + PM2).

### 2.2 Case 2

This female patient experienced the onset of seizures at 2 years of age, characterised by a fixed gaze, jaw clenching, cyanosis and tonic–clonic limb movements, with each episode lasting approximately 3–5 min. Seizures occurred every few months, and they were accompanied by a fever. At her first visit (3.5 years of age), she could sit but not walk. She had limited speech, hypotonia and dysmorphic features, including thick eyebrows, a narrow nose, micrognathia, large ear helices and slender fingers. Metabolic screening and routine biochemical examinations were unremarkable. MRI showed slightly wide sulci. EEG demonstrated generalised spike‐and‐wave complexes and polyspike‐and‐wave discharges, predominantly in the anterior head regions. Gesell's developmental schedules showed a profound developmental delay. After the initial sodium valproate (VPA) treatment, she had seizures about once every 6 months, most of which occurred following a fever. At age 5, her EEG captured two focal seizures predominantly originating from the right frontal, central and parietal regions (Figure S2A). Levetiracetam (LEV) was added to her treatment regimen. At 5 years and 8 months old, a follow‐up EEG revealed frequent focal seizures and electrographic seizures originating predominantly from the right central and occipital regions during both being awake and when asleep. The patient received methylprednisolone sodium succinate‐pulse therapy alongside continued VPA and LEV. She then remained seizure‐free for more than 1 year. At age 7, her seizures recurred as status epilepticus triggered by fever. Repeated EEGs revealed multifocal and diffuse spikes, spike‐and‐wave complexes and polyspike‐and‐wave discharges, as well as slow, sometimes rhythmic waves, which occurred primarily when the patient was asleep (Figure S2B). Brain MRI findings suggested cerebral atrophy and dilated supratentorial ventricles (Figure 1B). TPM was subsequently added to her regimen. She presented with short stature and microcephaly. At the last follow‐up at 9 years and 8 months of age, the patient has been seizure‐free for almost 2 years with continued VPA, LEV and TPM treatments. She could take a few independent steps with an unsteady gait, and the patient responded to names, but she was incapable of following commands. A splice‐site variant c.593‐3C>G in the ZEB2 gene (NM_014795.3) was identified near the splice acceptor region of Exon 6. Sanger sequencing confirmed that neither of the parents carried this variant, and this finding supported a de novo origin (Figure 2B). According to ACMG guidelines, this variant was classified as likely pathogenic (PS2 + PM2 + PP3).

## 3 Literature Review

A total of 12 cases were analysed, including 10 previously reported Chinese MWS children with epilepsy and the two cases reported in this study [4, 5, 6, 7]. Detailed clinical data are presented in Table 1.

**TABLE 1:** Phenotypic and genotypic characterisation of 12 MSW children with epilepsy.

ID	1	2	3^4^	4^4^	5^4^	6^4^	7^4^	8^5^	9^6^	10^6^	11^6^	12^7^
Gender	F	F	F	M	M	M	F	F	F	M	F	F
Onset age (month)	9	24	14	15	6	48	17			25	12	24
Last visit (month)	60	108	32	33	35	65	63	26	45	39		46
Abnormal MRI	+	+	−	+	+	−	−	+	+	+	+	+
Fever onset	−	+	+	−	−	+	+	+			−	+
Focal seizure	+	+	+	+		+	+			−		+
Absence seizure	−	−	−	−	−	−	−					
Other seizure	ES	GTCS			ES				ES/TCS	ES/TCS	GTCS	
Variant	c.530delC p.Pro177fs*34	c.593‐3C>G	c. 2712delT p.P906Lfs*24	c. 1027C>T p. Arg343*	c.2456C>G p.Ser819*	c. 1027C>T p. Arg343*	c.2761C>T p.Arg921*	c.756>A P.Y252X	c.2073G>A p.W691*,524	c.2073G>A p.W691*,524	c.2467C>T p. Q823*,392	c.164delC p.Pro55fs
ASM	ACTH/TPM	VPA/LEV/TPM	VPA	VPA	LEV/MPSS/TPM/VPA/LTG	LEV	VPA/LTG/ZNS/PER		LEV	VPA	VPA	
Seizure‐free	5 months	> 2 years	6 months	> 1 year	1 year	> 1 year	6 months			−	5 months	

*Note*: Cases 1 and 2 in the table are the cases in this study, and the remaining cases are from the labelled literature.

Abbreviations: −, no; +, yes; ACTH, adrenocorticotropic hormone; ASM, anti‐seizure medication; blank, unknown; ES, epileptic spasm; F, female; GTCS, generalised tonic–clonic seizure; LEV, levetiracetam; LTG, lamotrigine; M, male; MPSS, methylprednisolone sodium succinate; PER, perampanel; TCS, tonic–clonic seizure; TPM, topiramate; VPA, valproate; ZNS, zonisamide.

## 4 Discussion

There is speculation that the pathogenesis of epilepsy in MWS involves reduced numbers of gamma‐aminobutyric acid‐ergic (GABAergic) cortical interneurons and increased GABAergic subcortical and striatal interneurons [8, 9]. This neuronal imbalance disrupts corticosubcortical networks and may cause epileptic seizures [10, 11]. Seizures typically manifest during preschool years, although rare cases have been reported with neonatal and delayed‐onset epilepsy as late as 14 years after birth [12, 13]. Epilepsy in MWS can often manifest as febrile seizures, predominantly emerging within the first 2 years of life, and these episodes frequently occur during sleep. Among the 12 cases summarised in this study, 10 had documented ages of seizure onset, with 9 of them experiencing onset before the age of 3 years. Of the 12 cases, half of them presented with febrile seizures as the initial seizure type. Four cases experienced febrile seizures at ≤ 2 years of age.

The main epileptic phenotypes of MWS were focal and atypical absence seizures. Focal progression to bilateral tonic–clonic seizures, generalised tonic–clonic seizures, absence seizures and myoclonic seizures has been reported [1, 3, 14]. Among the 12 cases in this study, 7 (58.3%) exhibited focal seizures as the predominant epileptic phenotype. Atypical absence seizures demonstrated age‐related characteristics, emerging during the school‐age period and occurring in approximately 60% of cases [3, 15]. None of the cases reported the absence of seizures, which may be attributed to the young age of the patients and profound intellectual disability, obscuring seizure recognition. Careful observations, regular follow‐ups with EEGs and prolonged EEG‐monitoring periods are needed. Epileptic spasms were observed in a third of the cases, and these were rarely reported in the literature. This discrepancy may be related to limited sample sizes in existing studies. Thus, larger cohorts and long‐term follow‐ups are needed to clarify the seizure phenotypes in MWS.

The evolution of EEG patterns in MWS exhibits age‐dependent characteristics. EEG background activity during wakefulness and sleep may appear normal in early life. During the school‐age period, EEG background deteriorates, discharges increase, and non‐rapid eye movement abnormalities increase significantly, which constitutes the EEG pattern of electrical status epilepticus during sleep (ESES). The incidence of ESES can reach 35% in MWS. EEG abnormalities decrease significantly after the age of 13 years, corresponding to EEG evolution, and the frequency of seizures in children tends to decrease after puberty [15, 16]. In this study, both presented cases exhibited marked increases in epileptiform discharges during sleep on EEGs performed during the school‐age period. Case 2 developed electrical status ESES at approximately 5 years of age. Persistent significant epileptiform discharges were still observed on a follow‐up EEG at the age of 7 years.

There is no correlation between different degrees of brain malformations and MWS epilepsy [15]. All 12 cases in this study experienced epileptic seizures, with most sharing similar onset ages, seizure initiation patterns, and seizure types. Three cases showed no structural brain abnormalities, and 75% of them exhibited varying degrees of brain malformations, with Case 1 agenesis of the corpus callosum and Case 2 cerebral atrophy. Electroclinical evolution revealed the onset of seizures during early childhood, increased epileptiform discharges during sleep at school age, and a predominance of focal seizures as the main seizure type in later stages. Therefore, no significant correlation was found between brain structural abnormalities and epilepsy severity or phenotype, aligning with previous literature.

VPA is the most widely used and effective anti‐seizure medication (ASM) in MWS, followed by LEV. The efficacy of VPA may relate to its positive modulation of GABAergic activity in the cortex, which is deficient in these patients [15]. In MWS, ESES patterns may appear early and have a high discharge index, and they may persist chronically, which exerts adverse effects on cognition, and they should be treated appropriately [17]. Studies have shown that steroid treatments and surgery, which are not suitable for MWS, are the most effective therapies for ESES. Benzodiazepines may be suitable alternatives, but traditional ASMs demonstrate poor efficacy [18]. Carbamazepine, oxcarbazepine, and phenobarbital may exacerbate ESES, and they should be avoided. Among the 12 paediatric cases summarised in this study, 10 had documented ASM regimens. VPA was administered in seven cases, and three cases achieved seizure freedom for over 5 months after VPA monotherapy. These findings align with literature reports highlighting the broad application and efficacy of VPA in MWS. Other ASMs used alone or in combination included LEV, TPM, lamotrigine, zonisamide, and perampanel, with seizure‐free intervals ranging from 5 months to 2 years [19]. Methylprednisolone sodium succinate‐pulse therapy was initiated in Case 2 during the ESES episode, and it was successful in terminating the persistent epileptiform discharges observed on EEGs. Previous studies suggest that up to 25% of Caucasian patients experience refractory seizures, whereas only 19% of Chinese patients require long‐term ASMs, with the remainder achieving spontaneous remission without treatment [1, 20]. This discrepancy may arise from limited sample sizes, or it may reflect true ethnic variations, warranting further studies [1, 15].

## 5 Conclusion

MWS exhibits significant clinical heterogeneity and diverse manifestations, with no unified diagnostic criteria, making it prone to underdiagnosis. Clinicians should consider MWS in children presenting with distinctive facial features, multiple congenital anomalies, cognitive delay, and epilepsy initiating with febrile seizures, and pursue early genetic testing to confirm the diagnosis.

## Funding

This work was supported by financial grants from the Guangxi Science and Technology Program Project (Guike AB17195011), the Guangxi Medical and Health Appropriate Technology Development and Promotion Application Project (S2022039), the Guangxi Key Laboratory Open Project (GXWCH ZDKF‐2022‐16) and the High‐Quality Development Demonstration Project of the Maternal and Child Health Hospital of the Guangxi Zhuang Autonomous Region (GZLSF‐2).

## Ethics Statement

This study received ethical approval from the Ethics Committee of the Maternal and Child Health Hospital of Guangxi Zhuang Autonomous Region and was conducted in accordance with the Declaration of Helsinki (revised 2013). We confirm that we have read the journal's position on issues involved in ethical publication and affirm that this report is consistent with these guidelines.

## Consent

Written informed consent was obtained from the parents or legal representatives of the involved patients.

## Conflicts of Interest

The authors declare no conflicts of interest.

## Supporting information


**Figure S1:** EEG of Case 1 at 4 years and 10 months of age. Focal and diffuse slow waves, spike‐and‐wave complexes, and polyspike‐and‐wave discharges, predominantly in the anterior and posterior head regions, with marked activation during sleep.
**Figure S2:** EEG of Case 2. (A) EEG at 5 years of age showed multifocal and diffuse spikes, spike‐and‐wave complexes, polyspike‐and‐wave discharges, and slow, sometimes rhythmic waves, with sleep primarily. (B) EEG at 8 years of age showed multifocal and diffuse spikes, spike‐and‐wave complexes, polyspike‐and‐wave discharges, and slow, sometimes rhythmic waves, with sleep primarily.

## Data Availability

Data to support the findings of this study are included in the article and Supporting Information. Additional data may be available upon reasonable request.
