# Supplementary material for: Epilepsy in Chinese Children With Mowat–Wilson Syndrome: Two Case Reports and Literature Review
Source: J Paediatr Child Health. 2026 Mar 27;62(5):882–6. doi: 10.1111/jpc.70373 (PMC13172017; doi:10.1111/jpc.70373)
Supplement: Supplementary file 1 — Figure S1: EEG of Case 1 at 4 years and 10 months of age. Focal and diffuse slow waves, spike‐and‐wave complexes, and polyspike‐and‐wave discharges, predominantly in the anterior and posterior head regions, with marked activation during sleep. Figure S2: EEG of Case 2. (A) EEG at 5 years of age showed multifocal and diffuse spikes, spike‐and‐wave complexes, polyspike‐and‐wave discharges, and slow, sometimes rhythmic waves, with sleep primarily. (B) EEG at 8 years of age showed multifocal and diffuse spikes, spike‐and‐wave complexes, polyspike‐and‐wave discharges, and slow, sometimes rhythmic waves, with sleep primarily. [file JPC-62-882-s001.docx]

**SUPPLEMENTARY FIGURES**


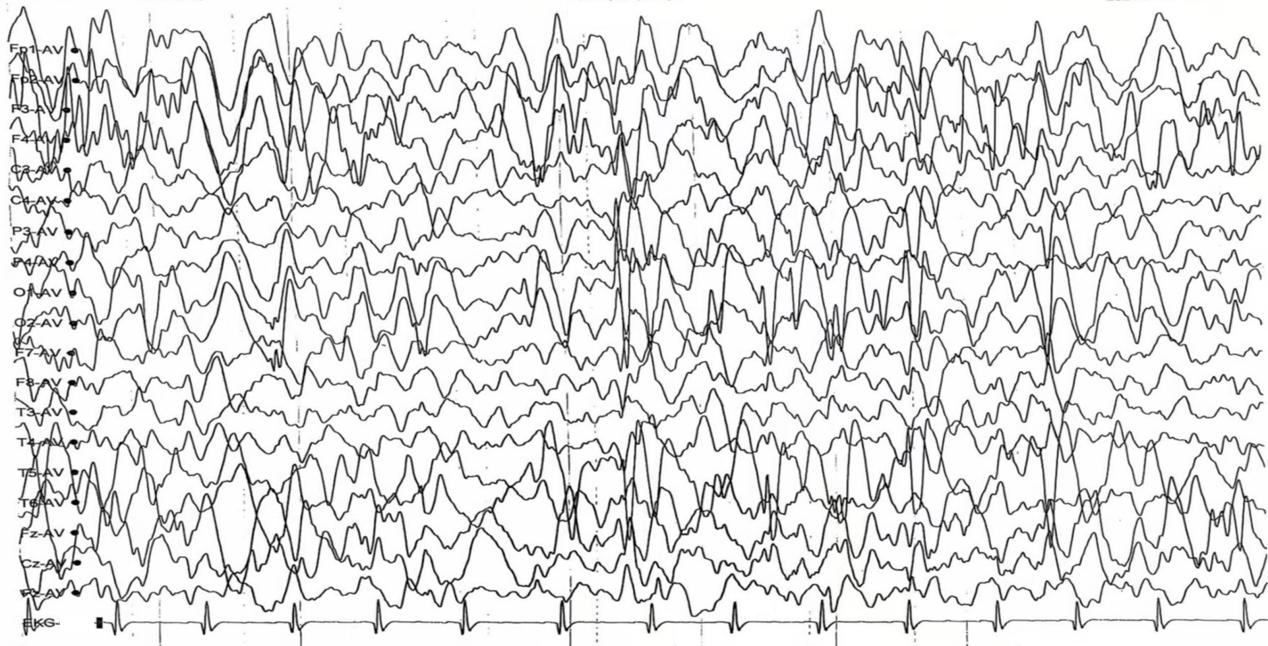


**Supplementary figure 1. EEG of Case 1 at 4 years and 10 months of age.** Focal and diffuse slow waves, spike-and-wave complexes, and polyspike-and-wave discharges, predominantly in the anterior and posterior head regions, with marked activation during sleep.


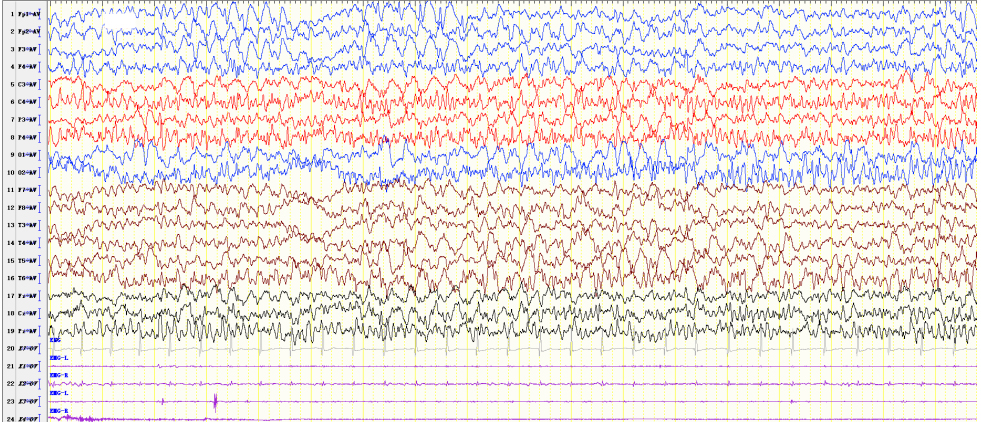


**A**


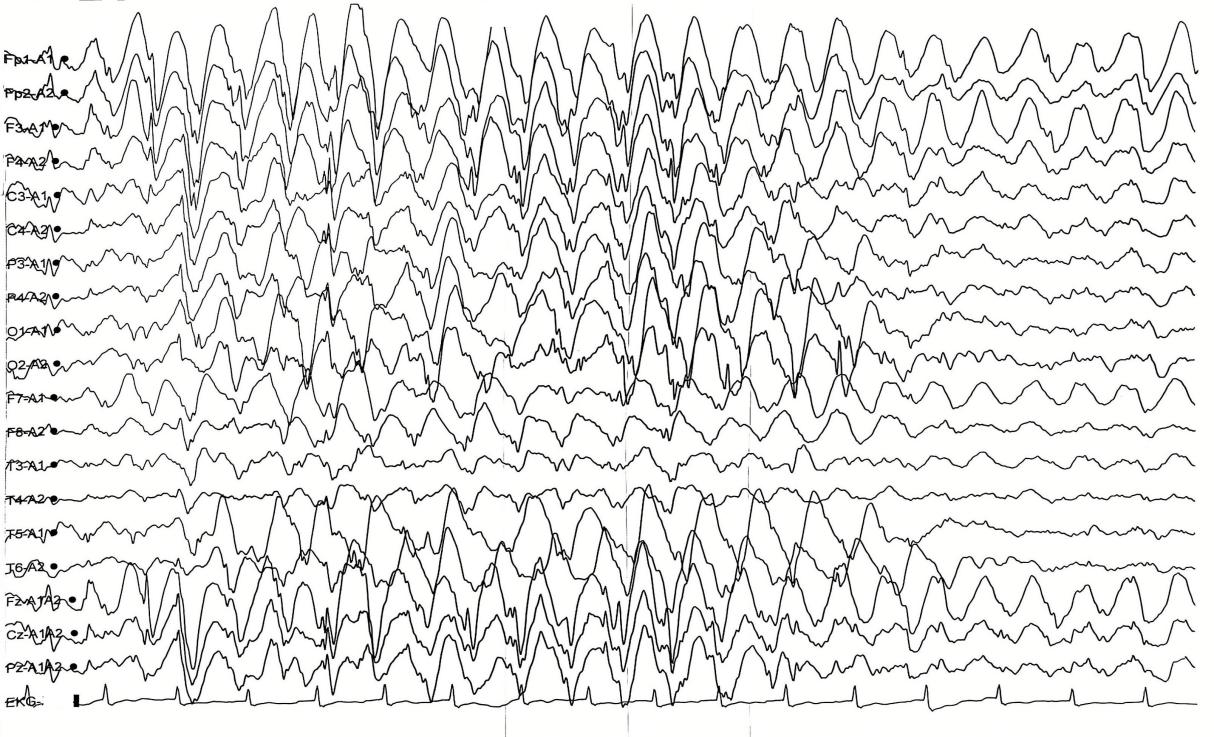


**B\**

**Supplementary figure 2. EEG of Case 2.** A: EEG at 5 years of age showed multifocal and diffuse spikes, spike-and-wave complexes, polyspike-and-wave discharges, and slow, sometimes rhythmic waves, with sleep primarily. B: EEG at 8 years of age showed Multifocal and diffuse spikes, spike-and-wave complexes, polyspike-and-wave discharges, and slow, sometimes rhythmic waves, with sleep primarily.
